# Impact of Innovation and Entrepreneurship Education in a University Under Personality Psychology Education Concept on Talent Training and Cultural Diversity of New Entrepreneurs

**DOI:** 10.3389/fpsyg.2021.696987

**Published:** 2021-07-29

**Authors:** Mengru Li, Tingting Wang, Yanhui Wu

**Affiliations:** ^1^College of Public Administration, Sichuan University, Chengdu, China; ^2^Faculty of Education and Human Development, The Education University of Hong Kong, New Territories, Hong Kong; ^3^Student Affairs Office, Beijing Normal University – Hong Kong Baptist University United International College, Zhuhai, China

**Keywords:** cultural diversity, talent training in colleges and universities, innovation and entrepreneurship education, entrepreneurship, university students’ innovation

## Abstract

The purpose of this study was to explore the cultivation of innovation and entrepreneurship of university students as well as its influence on talent training and cultural diversity. First, based on the combination of theoretical analysis and practical investigation, the entrepreneurship of college students and the basic situation of cultural diversity cultivation were discussed. The research objects involve students of different genders, grades, majors, and family backgrounds. Second, the reliability and validity of the scale were tested, and the survey data were analyzed after eliminating the invalid items. It is found that the average score of entrepreneurship of college students is 3.428; the score of innovation spirit dimension is 3.61, which is the highest in the dimensions; the score of forward-looking dimension is 3.25, which is the lowest; and the variance of leadership dimension is the lowest. The variance of the forward-looking dimension is the greatest. According to the analysis of the scores of different types of students, freshmen score lower in innovation dimension and challenge dimension, challenge spirit of female students is slightly higher than that of male students, and the leadership dimension score of science students is higher. Based on the survey results of cultural diversity training in colleges and universities, the average score is 3.343, the scores of course teaching and educational achievement are lower, and the score of cultural activity dimension is the highest. Liberal arts students have higher scores in cultural diversity, and senior students have low scores in cultural diversity cultivation due to the limited time. This study has been considered in a comprehensive manner. It provides a scientific theoretical basis for carrying out innovation and entrepreneurship education as well as cultural or nutritional activities in colleges and universities and thus has a significant practical value.

## Introduction

With the rapid social changes and growing international exchanges, the incoming of an era of knowledge-intensive economic development is expected. The culture exchange becomes more frequent, and the social pressure that college students bear is also increasing every year (Qian et al., 2018; Rogoza et al., 2018). In recent years, the policy of “mass entrepreneurship and innovation” brings the upsurge of entrepreneurship. With the continuous universalization of higher education in China, college students have become an important part of entrepreneurial groups under the influence of the entrepreneurial boom (Wu W. et al., 2019). Entrepreneurship is a kind of immaterial asset, which influences generations of young entrepreneurs (Wu Y. C. J. et al., 2019). Innovation is an essential part of entrepreneurship. Promoting and guiding entrepreneurship can relieve the unemployment of highly educated talents in the context of the global economic crisis, and the technological progress brought by entrepreneurship can provide new impetus for national development. Moreover, “entrepreneurship and innovation education” is also the educational content of ideological and political education in colleges and universities.

As social exchanges become more frequent, a large number of foreign cultures pour into China, triggering a series of new cultural trends (Chen, 2019). They not only enrich the treasure house of social culture but also broaden the vision of college students. However, they also cause some conflicts and collisions between foreign culture and Chinese culture, and some college students are affected by such negative thoughts as money worship, hedonism, and extreme individualism, leading to lack of ideals and beliefs, national self-confidence, and pride of some college students (Gou et al., 2020). Cultural diversity becomes a compulsory subject of ideological and political education in colleges and universities (Han et al., 2019). Then, how to carry out entrepreneurship and innovation education in college and university cultural diversity becomes a key issue. However, most previous studies often ignore the background of cultural diversity in the related research. Various problems caused by cultural diversity are failed to be addressed timely and properly, resulting in the disconnection of ideological and political education in colleges and universities till present (Zhang et al., 2018). Therefore, a great importance must be given to the combination of ideological and political education with entrepreneurship and innovation education, thereby promoting the full and comprehensive development of college students.

Based on the background of personality psychology, the theoretical basis of entrepreneurship, innovation education, and cultural diversity education were fully explored, and the basic situation and related characteristics of entrepreneurship cultivation and cultural diversity education of Sichuan University were analyzed by questionnaire survey. Theoretical analysis and case study were combined, which is an innovative point of this study. The basic information of the respondents and the entrepreneurship of Sichuan University and its cultural diversity course had been analyzed in detail. The main purpose of this study is to explore the current situation of entrepreneurship and innovation education in colleges and universities under the background of cultural diversity and to provide a scientific and effective reference for the reform and innovation of ideological and political education.

## Materials and Methods

### Role of Personality Psychology in Talent Training

The core of the personality psychology concept is the theory of personality characteristics. The foundation of personality structure is a trait, which mainly includes two types, namely, common trait and personal tendency (Meyer et al., 2019). Common traits are influenced by society, culture, and environment, and people with different cultural backgrounds have different common traits; the personal tendency is a unique trait of an individual, which is divided into a primary trait, core trait, and secondary trait (Wegmann et al., 2020). College students are in the stage of personality development and are exposed to the problems of self-identity confusion and uncoordinated interpersonal relationship. Furthermore, they are one of the groups who get in contact with new things fast; they are easy to feel the changing trend of the current social environment, which may have an impact on personality development in the process of socialization (Papageorgiou and Callaghan, 2018; Tajurahim et al., 2020). When college students are adversely affected by the social environment, the effects will cause conflicts with their inner values or social moral standards, and the phenomenon of self-inconsistency in the process of adapting to society appears, which easily leads to unbalanced development of personality (Vodă and Florea, 2019). As a result, it is necessary for college students to cultivate a healthy personality. In addition, students should be guided to integrate their personalities into the process of education and adjust the relationship between themselves and the ideal self (Sousa et al., 2018; Kulkarni et al., 2020).

It is commonly known that university is a key period of shaping and cultivating the personality and psychology of college students. Wu Y. C. J. et al. (2019) found that the social environment, school environment, family environment, and peer environment of college students can affect their personality psychology (Wu et al., 2018). Under the joint action of these factors, college students may have some social adaptation problems, and then there may be a series of personality disorders, which may bring about extremely serious consequences. Colleges and universities are the main places where students can study and live, and they also bear the main responsibilities and obligations to train talents and educate students. It is therefore concluded that education and life in colleges and universities are essential for talent training. Moreover, emphasis should be laid on the cultivation of personality and psychology of college students because of the pressure of adaption, interpersonal relationships, society, and employment. In the cultivation of personality psychology of college students, full consideration should be given to the cooperative education of theoretical knowledge and practical application of personality shaping. Meanwhile, in the process of ideological and moral education, the methods and innovative teaching modes should be improved. Wu and Song (2019) argued that in the teaching process, interaction and communication between lecturers and learners should be promoted and the active learning ability of students should be improved. In this context, the theoretical mechanism should be upgraded to help shape the personality of college students, establish the people-oriented education concept, and correctly guide the college students to develop sound psychological characteristics. Besides, a series of activities should be carried out to help students build their personality characteristics and meet their needs of interpersonal communication, employment, innovation, and entrepreneurship.

### Connotation of Entrepreneurship

Entrepreneurship is an important part of innovation and entrepreneurship education, and it is defined as the spiritual characteristics of entrepreneurs shown by their behaviors, thinking modes, and qualities (Ding, 2017). Therefore, entrepreneurship mainly refers to the firm belief and lofty value pursuit established by business operators in business practices, as well as the continuous innovative spirit and creativity. Entrepreneurship can be divided into innovation spirit, responsibility and dedication spirit, leadership and cooperation spirit, forward-looking spirit, and good faith spirit (Cadenas et al., 2020). Innovation spirit is the soul of entrepreneurship, and it is also an important component for promoting personal and social development. In terms of entrepreneurship of college students, the cultivation of innovation spirit is particularly important (Kanter et al., 2017; Mahendra et al., 2017).

### Embodiment of Cultural Diversity in Higher Education

Cultural diversity mainly means various forms of cultural expression of different groups and societies (Kisubi et al., 2021). The forms are reflected not only in different forms of expression, promotion, and inheritance of human cultural heritage but also in the ways and technologies of artistic creation, production, dissemination, sales, and consumption (Hitti et al., 2020). As time goes by, cultural diversity gradually becomes a power with the main culture as its body, multi-cultural as the complement, and shows up the trend of coexistence, blending, and common development (Wells, 2020). The ideological and political education in colleges and universities is a process of transmitting values and guiding ideological and political information to the educational objects after selection and design. However, with the development of cultural diversity and the deepening of reform and opening up, the ideological status of college students changes accordingly (Durkee et al., 2019). This requires the educators to make corresponding adjustments to the content and structure of higher education immediately so that the education can conform to the cultural development, capture the real cultural characteristics, and keep pace with the times (Mshigeni et al., 2020).

Based on cultural diversity, ideological and political educators and education objects develop more innovative ideas, which urge students to think independently and make choices. This education mode plays an important role in cultivating innovative talents, and it is also a key component of innovation and entrepreneurship education in colleges and universities (Maharaja, 2018). College students can experience the cultures of different countries, which is conducive to the renewal of ideas and the divergence of thinking as well as the realization of values of students. Cultural diversity is beneficial to the cultivation of the cultural consciousness of students, and it includes awakening and reflection, as well as cultural value judgment and choice (Campos and Kim, 2017). Without strong cultural awareness, there is no firm cultural confidence (Garriott et al., 2017). As a result, cultural diversity is closely related to ideological and political education in colleges and universities. In college education, much concern should be given to cultural diversity, together with practical activities and innovations, thereby making college education in line with the development of the times (Feng and Chen, 2020; Deng et al., 2021).

### Design and Analysis of Questionnaire

#### Design and Content of Scale

A questionnaire survey for freshmen to seniors in Sichuan University was conducted. In the design of the scale, the relevant research on entrepreneurship was referred by Obschonka et al. (2019), and the current situation of entrepreneurship and the cultivation of cultural diversity in colleges and universities were explored from the basic information of the research object, the analysis of entrepreneurship of students, and the cultivation of cultural diversity in a university.

The basic information involves gender, grade, subject distribution, and family situation (i.e., urban or rural family) of students. In terms of entrepreneurship, the questions were mainly drawn from the existing questionnaire of entrepreneurship. According to the status of Sichuan University, the scale was divided into four dimensions, namely, innovation spirit, challenge spirit, leadership ability, and forward-looking spirit. Four items were set in each dimension, with a total of 16 items. The 16 questions were designed based on the actual situation of students, involving the coping methods of difficulties of students in learning and entrepreneurship. The purpose is to analyze the specific performance of students in four dimensions, namely, innovation spirit, challenge spirit, leadership ability, and forward-looking spirit from the daily performance of students. The 5-point Likert scoring method was used for the evaluation index of the scale, in which “1” means “not consistent,” “2” means “relatively not consistent,” “3” means “consistent,” “4” means “relatively consistent,” and “5” means “totally consistent.”

The current situation of the cultivation of cultural diversity was investigated on the evaluation criteria of the effectiveness of ideological and political education in colleges and universities, from the perspective of cultural diversity proved in relevant studies. The investigation was carried out from four dimensions, namely, course teaching, cultural activities, extracurricular practice, and educational achievements. Each dimension includes four items, with a total of 16 items, mainly involving daily learning, practice, and leisure activities of students. These items were used to comprehensively analyze the current situation of cultural diversity education. The 5-point Likert scoring method was also used for the evaluation of the scale, with 1–5 points corresponding from “not consistent” to “totally consistent.”

#### Reliability and Validity Analysis of the Scale

The reliability and validity of the scale were analyzed before the data analysis. To make each item more accurately read, the items in the scale were numbered separately. The four dimensions of entrepreneurship were set as D1.1–D1.4, and the items in each dimension were labeled as 1, 2, 3, and 4; the four dimensions of cultural diversity cultivation were set as D2.1–D2.4, and the items were also labeled as 1, 2, 3, and 4. The Cronbach’s α coefficient is the main index of reliability analysis, and its value should be >0.7. The better the reliability of the scale, when the value is closer to 1.

## Results and Discussion

### Basic Statistics of Research Objects

**FIGURE 1:**
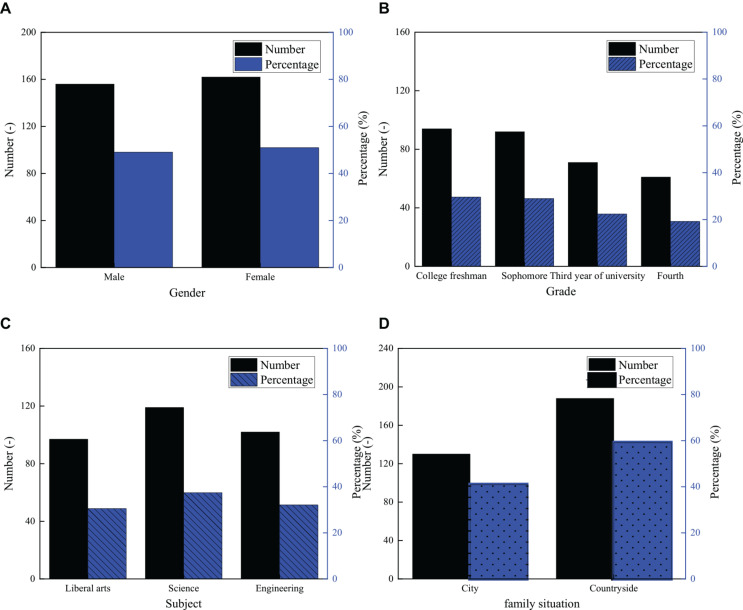
Basic information of the respondents. Statistical results of the respondents according to **(A)** gender, **(B)** grade, **(C)** subject, and **(D)** family situation are shown.

A total of 350 questionnaires were distributed to the students of Sichuan University, and 318 valid questionnaires were collected, with a recovery percentage of 90.86%. The basic information of the respondents is shown in Figure 1. Figures 1A–D show the statistical results of the respondents according to gender, grade, subject, and family situation.

Figure 1 shows that there are 156 male students, accounting for 49.06% of the total number, and 162 girls, accounting for 50.94%. The gender proportion of the respondents surveyed is almost the same. Regarding the grade, most of them are freshmen, and the number is 94, accounting for 29.56%; the number of seniors is 61, which is the fewest, accounting for 19.18%. Among the students surveyed, 97 students are in arts, accounting for 30.50%, 119 students are in science, with a ratio of 37.42%, and 102 are engineering students, accounting for 32.08%. According to the family situation of the students, 130 students are from cities, occupying 40.88%, while 188 students are from rural areas, accounting for 59.12%. The number of rural students is slightly higher than that of urban students. The results show that the proportion of the males and the females in this survey is basically the same, but their family situation is similar and their professional background is diverse, which proves that the research object covers different types of college students. It is worth noting that freshmen account for a large proportion in this study, and they are also the main audience of innovation and entrepreneurship education in colleges and universities. Therefore, the results based on this survey sample have a certain practical value.

### Reliability and Validity of the Scale

**FIGURE 2:**
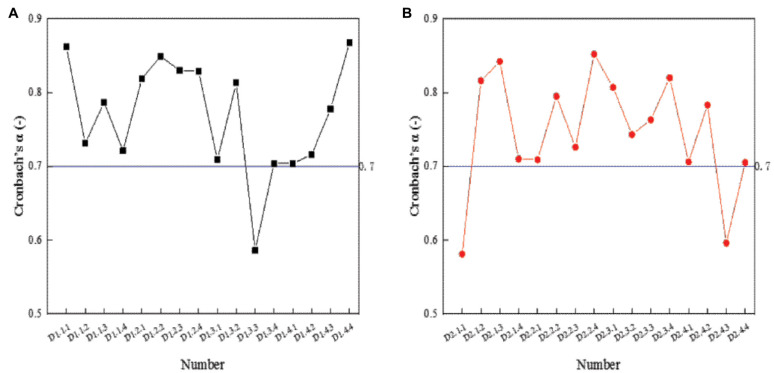
Reliability test results of the scale. **(A)** Reliability analysis results of entrepreneurship. **(B)** Reliability analysis results of cultural diversity cultivation.

The reliability and validity of the questionnaire are shown in Figure 2. Figure 2A shows the reliability analysis results of entrepreneurship, and Figure 2B shows the reliability analysis results of cultural diversity cultivation:

In Figure 2, the Cronbach’s α coefficients of D1.3.3, D2.1.1, and D2.4.3 are <0.7, while the Cronbach’s α coefficients of the rest items are >0.7. The results show that the three items do not pass the reliability test, and the data of the three need to be eliminated.

**FIGURE 3:**
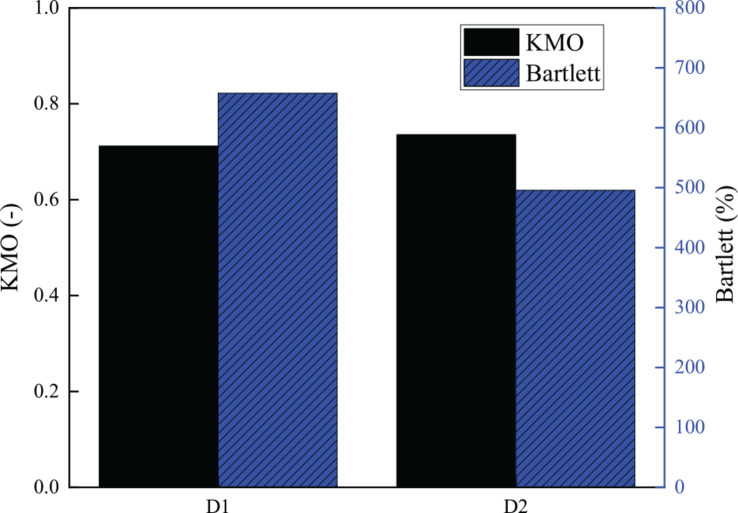
Validity test results of the scale.

In the validity test, Kaiser–Meyer–Olkin (KMO) and Bartlett hemispheric physical examination were used as evaluation indexes, and the value of KMO was required to be >0.7. SPSS 25.0 (IBM, New York, United States) data analysis software was used for calculation. The specific results of the validity test are shown in Figure 3.

According to the validity test results of the scale, the KMO value of entrepreneurship is 0.712, and the KMO value of cultural diversity is 0.736. The values are >0.7, indicating that the scale is valid in the validity test.

### Current Situation of Entrepreneurship of College Students

**FIGURE 4:**
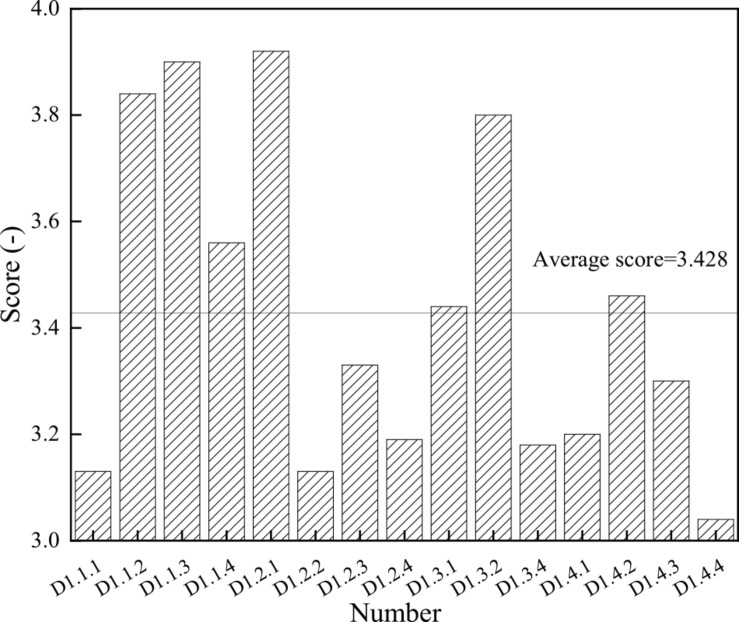
Scores of entrepreneurship of college students.

**FIGURE 5:**
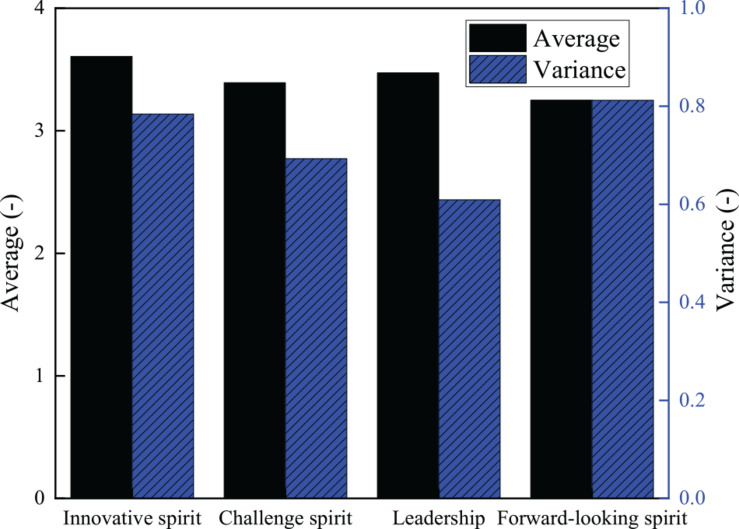
The score of each dimension of the research object.

Based on the collection and collation of questionnaire survey data, the specific situation of entrepreneurship of college students is obtained. Figure 4 shows the score of each item, and Figure 5 shows the average score and variance of each dimension.

Figure 4 has 15 effective items: D1.2.1 is the first item of the challenging spirit dimension and scores 3.92, and it is the highest; D1.4.4 is the fourth item of the challenging spirit dimension and has the lowest average score of 3.04. The average score of the spirit of college students is 3.428, and eight items are below the average score. Figure 5 indicates that among the four dimensions of entrepreneurship, the average score of innovation spirit is 3.61 and the variance is 0.784, the average score of challenge spirit is 3.39 and the variance is 0.693, the average score of leadership is 3.47 and the variance is 0.609, and the average score of forward-looking spirit is 3.25 and its variance is 0.812. The above results show that in the four dimensions of entrepreneurship, the average score of innovation spirit is the highest, while the average score of forward-looking spirit is the lowest. The results of variance analysis show that the variance of leadership is the smallest and that of forward-looking spirit is the largest. Therefore, it can be concluded that the students of the university have innovative spirit, but they lack forward-looking spirit. The score gap between the respondents is also large. The main reason for this phenomenon is that most college students spend much time on their studies and have no time to do extracurricular practice, lacking the correct positioning to the market and entrepreneurial opportunities; besides, the lack of social experience and related theoretical knowledge of college students leads to the low score of the forward-looking spirit of college students. In view of the above situation, colleges and universities need to cultivate the forward-looking spirit of college students. The above Figures 4, 5 shows that the innovation and entrepreneurship course of Sichuan University has a positive impact on college students. It improves the innovation spirit and entrepreneurship of students and helps them take part in employment and entrepreneurial activities more actively.

**FIGURE 6:**
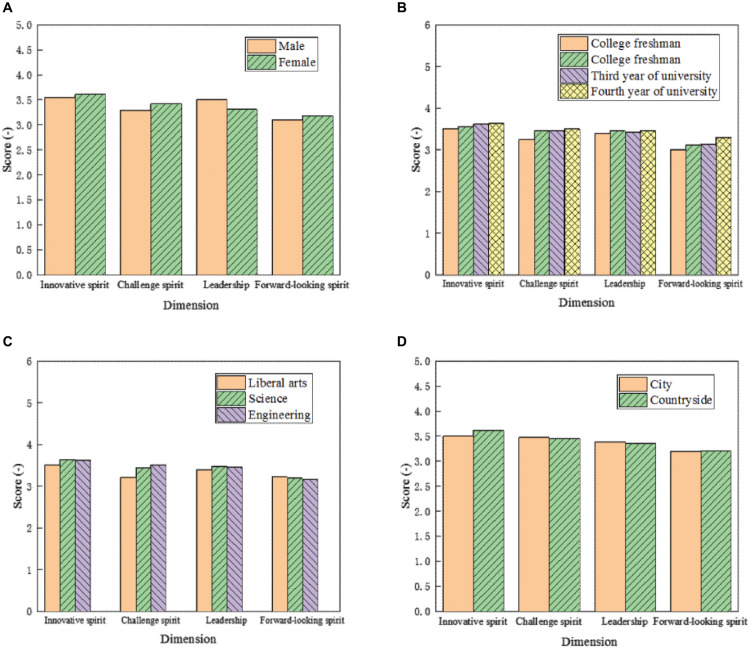
Scores of entrepreneurship of different students. The scores of entrepreneurship of students with different **(A)** genders, **(B)** grades, **(C)** subjects, and **(D)** family situations are shown.

To further improve the grades of entrepreneurship of different types of students, more targeted cultivation of entrepreneurship should be carried out. Based on the survey data, the scores of entrepreneurship of students with different genders, grades, subjects, and family situations were explored, as shown in Figures 6A–D.

The statistical analysis results in Figure 6 demonstrates that the scores of the dimension of innovation spirit between male and female students are slightly different; the scores of juniors and seniors are slightly higher than those of the freshmen and sophomores. The scores of urban students are slightly higher than those of rural students, while the scores of science students in the dimension are higher than those of other subjects. The score of female students in the challenge spirit dimension is slightly higher than that of male students, and the score of freshmen is significantly lower than that of other grades. The scores of engineering students are the highest, and the difference between urban students and rural students is not significant. In the leadership dimension, the score of male students is slightly higher than that of female students, the scores of students with different grades are low, the score of science students is higher, and the score of urban students is slightly higher than that of rural students. In terms of the forward-looking spirit dimension, except for seniors, there is no significant difference between the other types of students. The main reason for this phenomenon is that the senior students are facing the pressure of employment, and some college students choose to start their own businesses. Therefore, they usually pay attention to the research on innovation and entrepreneurship to have rich experience and more knowledge. In this case, the score of the forward-looking spirit of senior students is significantly improved.

### Cultivation Status of Sichuan University

**FIGURE 7:**
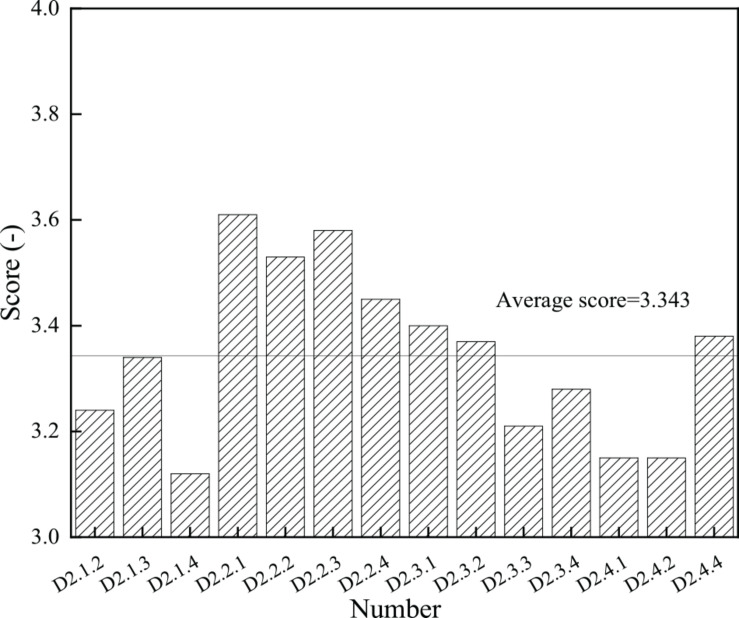
Scores of each item in cultural diversity cultivation.

**FIGURE 8:**
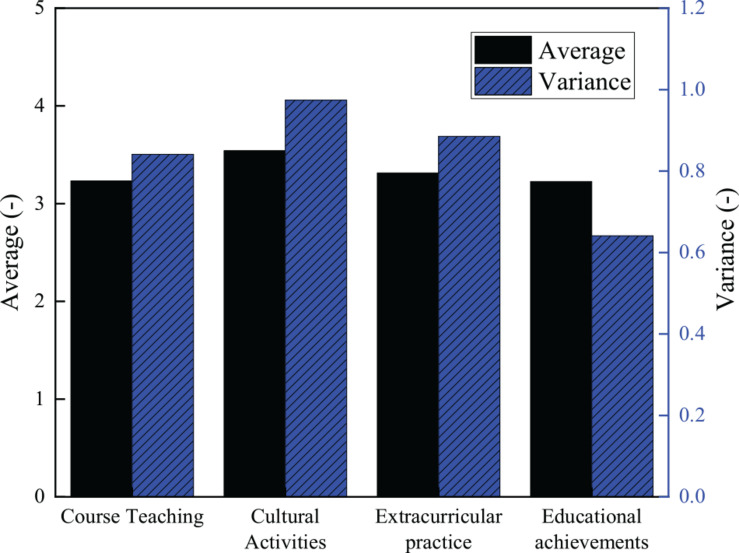
Scores of each dimension in cultural diversity cultivation.

A statistical analysis of the results of the cultural diversity survey was conducted. The score of each item of cultural diversity training is shown in Figure 7, and the total analysis result of each dimension is shown in Figure 8.

Figure 7 indicates that the highest score is 3.61 and corresponds to item D2.2.1, which is the first item in the cultural activity dimension. D2.4.1 and D2.2.2 score the lowest score of 3.15, which are the second and third items of the dimension of educational achievements. The average score of cultural diversity is 3.343, and there are six items below the average, mainly concentrated in the dimension of course teaching and educational achievements. The results of variance analysis show that the gap between the students in cultural activities is the largest, while the gap in educational achievements is the smallest. Therefore, the cultural activities should be strengthened in the follow-up cultural diversity education.

Figure 8 proves that through the deep analysis of the four dimensions, the average score of course teaching is 3.233, and its variance is 0.841; the average score of cultural activity dimension is 3.542, and the variance is 0.974; the average score of extracurricular practice is 3.315, and the variance is 0.885; and the score of educational achievement dimension is 3.227, with the variance of 0.641. The survey data show that the cultivation effect in cultural activities and extracurricular practice of Sichuan University is excellent, but the course teaching and the educational achievements score low. This indicates that colleges and universities need to fully implement targeted cultural diversity training courses, increasing the achievements of cultural diversity.

**FIGURE 9:**
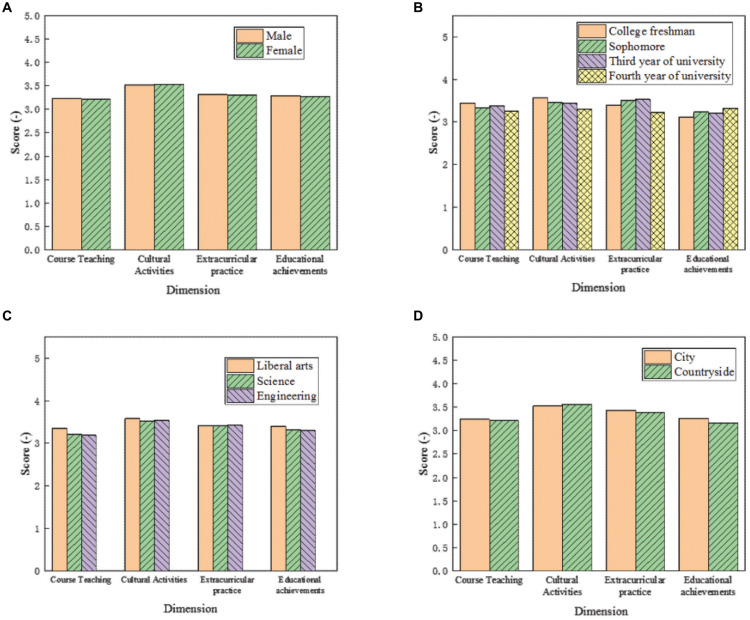
Cultural diversity cultivation of different types of college students. The scores of cultural diversity cultivation of students with different **(A)** genders, **(B)** grades, **(C)** subjects, and **(D)** family situations are shown.

The basic situation of cultural diversity cultivation of different types of college students is shown in Figure 9. Figures 9A–D correspond to the scores of cultural diversity cultivation of students with different genders, grades, subjects, and family situations, respectively.

The scores of the items in the questionnaire among different types of students were analyzed as follows: in terms of gender, there is a small gap between males and females in the four dimensions of the cultivation of cultural diversity; regarding the grade, freshmen score the highest in the four dimensions of the cultivation of cultural diversity, followed by sophomores and juniors, and seniors score the lowest, which is in line with the general law of teaching and training in Sichuan University. Since most of the time and energy of seniors are used in postgraduate entrance examination and employment, they spend less time on cultural diversity courses, especially on cultural activities and practical activities; regarding the major, the scores of liberal arts students are higher than those of science students and engineering students. This is mainly because the curriculum of liberal arts students is relatively rich and that of engineering students is more professional. Therefore, liberal arts students score significantly higher in the extracurricular course of cultural diversity. With regard to family status, the scores of urban students are higher than those of rural students in educational achievements, and there is no obvious difference in the rest.

## Conclusion

In this study, the students of different genders, grades, subjects, and family situations in Sichuan University were explored. A questionnaire survey was conducted to collect and sort out the data, and the questionnaires based on the entrepreneurship and cultural diversity education of students were designed. After the reliability and validity test of the scale, three invalid items were excluded. The results of effective items were analyzed and processed, and the conclusions were drawn as follows: the overall average of entrepreneurship is 3.428, the score of challenge spirit is 3.39, the score of leadership is 3.47, the score of innovation spirit is 3.61, and the score of forward-looking spirit is 3.25. The variance of the leadership dimension is the least, which is 0.609, and the score of the forward-looking spirit dimension is very low for 0.812. Therefore, the university should focus on the cultivation of the forward-looking spirit. Among the students of different types, the score of challenge spirit dimension of female students is slightly higher than that of male students, and the engineering students score the highest; the score of freshmen is lower in the challenging spirit, innovation spirit, and forward-looking spirit dimensions. The average score in the cultural diversity training of colleges and universities is 3.343, and the score of course teaching and educational achievement dimension is lower, with the scores of 3.233 and 3.227, respectively; the highest score of cultural activity dimension is 3.524. According to the scores of different subjects, liberal arts students generally score higher, and senior students generally score lower. Therefore, the school can strengthen the training of cultural diversity courses and emphasize the cultural diversity education of science and engineering students.

This research content is based on the reality of innovation and entrepreneurship education in colleges and universities, and the investigation content is comprehensive. The situation of different types of students is analyzed pertinently, which provides a scientific and effective reference for optimizing the innovation and entrepreneurship education mode. However, due to the various factors, the number of subjects in this study is limited, and the research on students of different natures is not involved. These problems should be addressed in the follow-up studies. Besides, there is no comprehensive and long-term research on the cultivation of innovation and entrepreneurship of college students, which will be the future focus.

## Data Availability Statement

The raw data supporting the conclusions of this article will be made available by the authors, without undue reservation.

## Ethics Statement

The studies involving human participants were reviewed and approved by the Sichuan University Ethics Committee. The patients/participants provided their written informed consent to participate in this study. Written informed consent was obtained from the individual(s) for the publication of any potentially identifiable images or data included in this article.

## Author Contributions

All authors listed have made a substantial, direct and intellectual contribution to the work, and approved it for publication.

## Conflict of Interest

The authors declare that the research was conducted in the absence of any commercial or financial relationships that could be construed as a potential conflict of interest.

## Publisher’s Note

All claims expressed in this article are solely those of the authors and do not necessarily represent those of their affiliated organizations, or those of the publisher, the editors and the reviewers. Any product that may be evaluated in this article, or claim that may be made by its manufacturer, is not guaranteed or endorsed by the publisher.
